# Endogenous tPA levels: A biomarker for discriminating hemorrhagic stroke from ischemic stroke and stroke mimics

**DOI:** 10.1002/acn3.52197

**Published:** 2024-09-10

**Authors:** Melissa Jauquet, Pierre Gagnepain, Estelle La Porte, Audrey M. Thiebaut, Ambre Rochey, Helene Legros, Baptiste Laine, Marion Berthelot, Valerie Roussel, Joan Montaner, Barbara Casolla, Denis Vivien, Eloise Lemarchand, Richard Macrez, Benoit D. Roussel

**Affiliations:** ^1^ Normandie University, UNICAEN, INSERM, UMR‐S U1237, Physiopathology and Imaging of Neurological Disorders (PhIND), Institute Blood and Brain @Caen‐Normandie (BB@C), GIP Cyceron Caen France; ^2^ Normandie University, UNICAEN, PSL Research University, EPHE, INSERM, U1077, CHU de Caen, GIP Cyceron, Neuropsychologie et Imagerie de la Mémoire Humaine Caen 14000 France; ^3^ Centre de Ressources Biologiques InnovaBIO Caen University Hospital Caen France; ^4^ Department of Clinical Research Caen University Hospital Caen France; ^5^ Department of Neurology Hospital Universitario Virgen Macarena Sevilla Spain; ^6^ UR2CA‐URRIS, Stroke Unit, CHU Pasteur 2 Nice Cote d'Azur University Nice France; ^7^ Department of Emergency Medicine Caen University Hospital Caen France

## Abstract

**Objective:**

Stroke is the leading cause of death and disability. Timely differentiation between ischemic stroke, hemorrhagic stroke, and stroke mimics is critical for tailored treatment and triage. To accelerate the identification of stroke's subtype, we propose to use the levels of circulating tPA as a biomarker.

**Methods:**

Biostroke is an observational study performed at the Caen Hospital. We quantified tPA levels in 110 patients with ischemic strokes, 30 patients with hemorrhagic strokes, and 67 stroke mimic patients upon their arrival at the emergency. Two logistic regression models were formulated: one with parameters measurable in an ambulance (Model A) and one with parameters measurable at the hospital (Model H). These models were both tested with or without plasma tPA measurements. Our initial assessment involved evaluating the effectiveness of both models in distinguishing between hemorrhagic strokes, ischemic strokes, and stroke mimics within our study cohort.

**Results:**

Plasmatic tPA levels exhibit significant distinctions between hemorrhagic, ischemic, and mimic stroke patients (1.8; 2.5; 2.4 ng/mL, respectively). The inclusion of tPA in model A significantly enhances the classification accuracy of hemorrhagic patients only, increasing identification from 0.67 (95% CI, 0.59 to 0.75) to 0.78 (95% CI, 0.7 to 0.85) (*p* = 0.0098). Similarly, in model H, classification accuracy of hemorrhagic patients significantly increased with the addition of tPA, rising from 0.75 (95% CI, 0.67 to 0.83) without tPA to 0.86 (95% CI, 0.81 to 0.91) with tPA (*p* = 0.024).

**Interpretations:**

Our findings underscore the valuable role of tPA levels in distinguishing between stroke subtypes.

## Introduction

Stroke is the second leading cause of death and the primary cause of adult disability.^1^ Of all strokes, 87% are ischemic, and 13% are hemorrhagic.^2^ The emergency is to distinguish between these two subtypes. Many studies failed identifying clinical features to help differentiating stroke subtypes and false‐positive cases commonly referred to as stroke mimics. The incidence of stroke mimics is approximated to range between 20 and 50%, with variations influenced by the context of evaluation, such as whether it occurs in the emergency setting or within a dedicated stroke unit.^3^, ^4^ In acute ischemic stroke, the primary objective is to restore blood flow. Currently, two acute treatments are available: thrombolysis, which can be achieved using tissue‐type plasminogen activator (tPA) or tenecteplase (TNK)^5^ and mechanical thrombectomy.^6^ The differential diagnosis of ischemic and hemorrhagic stroke typically relies on clinical criteria and the analysis of a brain imaging performed using a computed tomography (CT) scan or magnetic resonance imaging (MRI). The brain imaging is mainly useful to eliminate a diagnosis of hemorrhagic stroke. While CT scans are widely accessible, they often yield normal results during the acute phase of the disease. Conversely, MRI is more sensitive in detecting acute ischemic events but may present challenges due to a limited availability. Despite evaluation by expert stroke specialists, misdiagnosis of stroke mimics also occurs in clinical practice.^7^ Given these challenges, the development of a blood‐based biomarker for stroke diagnosis holds significant promise, rapidly feasible at the arrival at the emergency unit and possibly in the ambulance.

Interestingly, in order to protect the body against intravascular thrombosis, endothelial cells release tPA acutely.^8^ Accordingly, human experiments conducted on the forearm have demonstrated that this release of tPA can reach levels of up to 4.5 μg/min after the application of a tourniquet, sustaining these levels for extended periods.^9^ Similarly, it is well admitted that an increased shear stress in the brain circulation also promotes the release of tPA.^10^ For instance, while venous shear stress does not lead to an increase in the release of tPA, arterial shear stress results in a 2.1‐fold increase in its release.^10^

Circulating tPA has been utilized as a biomarker for cardiovascular diseases, with a positive correlation observed between high levels of plasmatic tPA and coronary heart diseases.^11^ In stroke patients, levels of circulating tPA are significantly elevated after stroke.^12^, ^13^ They found higher levels of tPA in ischemic stroke patients compared with the control group. However, this study demonstrates the prognostic utility of tPA as a biomarker: Due to the timing of blood collection (up to 7 days following the event), conclusions regarding its use as a diagnostic biomarker during the acute phase cannot be drawn.^13^

Based on these observations, we aimed to determine whether the concentration of total plasmatic tPA levels could serve as a valuable biomarker for distinguishing between hemorrhagic stroke, ischemic stroke, and stroke mimics. We then used this blood‐based biomarker as an adjunct parameter in either an ambulance or a hospital made scoring to improve triage of patients.

## Material and Methods

### Study design and patient selection

Patients of the study are from Biostroke cohort, an observational, prospective, monocentric study performed at the emergency department in collaboration with the Center of Biological Resources (CRB InnovaBIO) of the Centre Hospitalo‐Universitaire de Caen‐Normandie, France, since 2018. Every patient gave their consent according to the Declaration of Helsinki, and the bio‐collection has been approved by ethics committees (CPP number: DC 2014‐2154; French Ministry of Health). All patients or relatives receive information at inclusion. Biostroke includes all patients at their arrival at the emergency service with an age ≥18; with stroke suspicion; with a time from symptom onset to blood samples collection ≤48 h and prior to any treatment. Patients with a history of chronicle inflammatory pathology, cancer, or hemostasis trouble were excluded. After inclusion, clinical and radiological data were collected into standardized forms at hospital admission. All stroke diagnoses were reviewed and confirmed by a certified neurologist.

**Figure 1:**
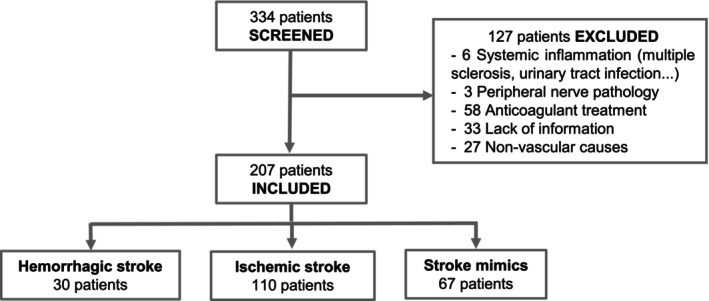
Flow chart of the selected patients within the Biostroke collection. Flow chart of patients selected for the study.

For the present study, we include all patients of the cohort, from 2018 to 2022, with a final diagnosis of ischemic stroke, hemorrhagic stroke, and stroke mimics. TIA patients, patients with pathologies known to modify tPA levels (i.e., systemic inflammation; cancer), patients with anticoagulant treatments, and patients in which all the information were not available were excluded (Fig. 1). Symptom's severity in Biostroke was assessed with the National Institutes of Health Stroke Scale (NIHSS) score.

### Blood samples collection

Blood was drawn on admission at the emergency department before any treatment. Samples are constituted from remaining blood in citrate tubes sampled to routine hemostasis examination of patients admitted with a suspected stroke diagnosis. Blood samples are centrifuged at 2300 *g* for 11 min at room temperature then a second time after a passage in the automat, with the same parameters. Plasma is collected for the routine examination of patient, and the rest is frozen at −80°C in the biobank until biomarker measurement.

### Biomarker measurement

Plasma antigenic tPA measurement was performed by enzyme linked immunosorbent assay from Molecular Innovation (Human Total tPA ELISA Kit, Innovative Research). All assays were performed in duplicate according to the manufacturer's instructions.

### Statistical analysis

All univariate analyses were conducted using RStudio, version 4.1.2. Categorical variables were presented as numbers (percentages), while continuous variables were expressed as mean ± SD. We performed comparisons between each group, utilizing the chi‐square test for categorical variables and, depending on the data distribution assessed by the Shapiro–Wilk test, either the Student *t*‐test or the Mann–Whitney U test for continuous variables. A significance level of *p* < 0.05 was considered statistically significant. To estimate the minimal sample size to detect the presence of tPA difference, we conducted a priori power analysis. This analysis revealed that a *N* = 25 was sufficient to detect group differences in the level of tPA with a statistical power of 80%. G*Power (version 3.1.9.7) was utilized for these power calculations. The utility of tPA as a potential biomarker was assessed by comparing the area under the curve (AUC) of our logistic regression models. The rationale of the prespecified models was to adjust for known predictors of stroke. Those included age (continuous), sex (yes or no), baseline RACE score (continuous) for the “ambulance model”; and age (continuous), sex (yes or no), baseline NIHSS score (continuous), and systolic blood pressure (continuous) for the “hospital model.” These two models are detailed in the result section. Statistical comparison of AUC values was performed by generating model predictions through bootstrapping and computing corresponding AUC values 5000 times. Model predictions were additionally computed using a 10‐fold cross‐validation with 3 repetitions and stratified sampling. These analyses were performed using a combination of *glmfit.m*, *perfcurve.m* and *cvpartition.m* in Matlab (the MathWork).

## Results

### Patient selection

A total of 207 patients were enrolled in this study from 2018 to January 2022, originating from the Biostroke study conducted at Caen Normandie University Hospital in France. Among these individuals, 110 were confirmed to have experienced an ischemic stroke, 30 were confirmed to have had a hemorrhagic stroke, and 67 presented with conditions that mimicked stroke symptoms (Fig. 1). Of the total stroke patients (*n* = 140), 79% were diagnosed with ischemic strokes (*n* = 110), while 21% had hemorrhagic strokes (*n* = 30); and in the entire cohort of included patients (*n* = 207), 32% were categorized as stroke mimics (*n* = 67). A descriptive analysis of the whole cohort and the differences between groups is detailed in Table 1.

**Table 1:** Descriptive analysis of hemorrhagic, ischemic stroke, and stroke mimics patients and differences between groups.

Characteristic	Hemorrhagic stroke (1)	Ischemic stroke (2)	Stroke mimics (3)	*p* value [95% CI] (1) vs (2) (2) vs (3) (1) vs (3)
Sample size	30	110	67	
Age, median (IQR)	73 (64–82)	74 (63–84)	62 (50–76)	0.6504 [−4.57 to 7.30] 0.0007* [4.00 to 15.9] 0.2657 [4.95 to 17.5]
Sex (% female)	14 (47)	46 (42)	41 (61)	0.1014 [−0.01 to 0.27] 0.01898* [−0.33 to 0.71] 0.001249* [−0.15 to −0.46]
Arterial hypertension (%)	21 (70)	68 (62)	25 (37)	0.5409 [−0.01 to 0.09] 0.0026* [0.08 to 0.38] 0.005783* [0.46 to 0.17]
Hypertension drug (%)	16 (53)	68 (62)	21 (31)	0. 5283 [−0.11 to 0.24] 0.0001581* [0.14 to 0.43] 0.06656 [−0.01 to 0.41]
Atrial fibrillation (%)	0 (0)	18 (16)	1 (1.5)	0.03885* [0.14 to 0.35] 0.004377* [0.21 to 0.52] 1 [−0.72 to 0.09]
SBP in mmHg, mean (SD)	170 (25.6)	155 (23.6)	144 (24.7)	0.01078* [3.58 to 25.7] 0.004211* [3.74 to 19.5] <0.0001* [14.5 to 38.1]
DBP in mmHg, median (IQR)	86 (75–92)	83 (70–98)	76 (68–91)	0.3126 [−5.53 to 5.47] 0.09585 [13.9 to 37.9] 0.08806 [−0.99 to 9.99]
Glycemia in g/L, median (IQR)	1.3 (1.1–1.6)	1.1 (1.0–1.4)	1.1 (0.9–1.2)	0.2104 [−0.01 to 0.29] 0.02587 [0.01 to 0.18] 0.003019* [0.07 to 0.33]
Current or past tobacco use (%)	7 (23)	46 (42)	20 (30)	0.1014 [−0.01 to 0.28] 0.1508 [−0.36 to 0.27] 0.6768 [−0.26 to 0.32]
Alcohol (%)	5 (17)	33 (30)	12 (18)	0.2209 [−0.04 to 0.26] 0.1066 [−0.02 to 0.31] 1 [0.74 to 0.58]
Migraine (%)	0 (0)	2 (1.8)	7 (10)	1 [−0.07 to 0.50] 0.02911* [−0.76 to −0.08] 0.1575 [−0.51 to −0.15]
Diabetes mellitus (%)	3 (10)	11 (10)	6 (9.0)	1 [−0.23 to 0.23] 1 [−0.24 to 0.29] 1 [−0.34 to 0.30]
Antidiabetic drug (%)	3 (10)	8 (7.2)	6 (9.0)	0.9129 [−0.39 to 0.26] 0.9083 [−0.37 to 0.25] 1 [−0.34 to 0.30]
Dyslipidemia (%)	10 (33)	35 (32)	18 (27)	1 [−0.17 to 0.15] 0.5971 [−0.51 to −0.15] 0.6838 [−0.12 to 22]
Dyslipidemia treatment (%)	7 (23)	28 (25)	14 (21)	1 [−0.15 to 0.19] 0.6105 [−013 to 0.23] 0.9978 [−0.23 to 0.30]
Coronary artery disease (%)	1 (3.3)	12 (11)	2 (3.0)	0.3615 [−0.05 to 0.35] 0.108 [0.02 to 0.49] 1 [−0.54 to 0.59]
Previous ischemic stroke (%)	2 (6.6)	6 (5.4)	7 (10)	1 [−0.38 to 0.31] 0.3482 [−0.50 to 0.14] 0.83 [−0.45 to 0.25]
Previous hemorrhagic stroke (%)	3 (10)	0 (0)	0 (0)	0.008253* [−1.00 to −0.56] ‐ 0.04605* [0.45 to 0.97]
mRS score of >2 (%)	2 (6.6)	11 (10)	4 (6.0)	0.8393 [−0.18 to 0.32] 0.5122 [−0.16 to 0.39] 1 [−0.34 to 0.30]
NIHSS score, median (IQR)	15 (3.5–21)	5 (1.0–13)	0 (0–3)	0.0175* [0.81 to 8.28] <0.0001* [2.01 to 6.00] <0.0001* [5.99 to 16.9]
RACE score, median (IQR)	6 (3.5–8)	4 (2–6)	2 (0–2)	0.2664 [−2.00 to 0.00] <0.0001* [2.01 to 3.99] <0.0001* [2.01 to 5.99]
Time from symptom onset to hospital admission in h, median (IQR)	1.3 (1.7–2.3)	1.2 (1.2–2.9)	1.3 (1.3–3.1)	0.6429 [−0.50 to 0.28] 0.3509 [−0.19 to 0.51] 0.2158 [−0.15 to 0.70]

Baseline characteristics of patients from Biostroke collection, and comparison between hemorrhagic stroke (1) versus ischemic stroke (2); ischemic stroke (2) versus stroke mimics (3); and hemorrhagic stroke (1) versus stroke mimics (3). Data are presented as n (%) for categorical variables and as median (IQR) or mean (SD) for continuous variables. Chi‐square test for categorical variables, Student *t* or Mann–Whitney *U* test for continuous variables.

CI, confidence interval; DBP, diastolic blood pressure; IQR, interquartile range; NIHSS, National Institutes of Health Stroke Scale; RACE, Rapid Arterial oCclusion Evaluation; SBP, systolic blood pressure; SD, standard deviation.

* *p* < 0.05.

### Measures of plasmatic tPA levels

**Figure 2:**
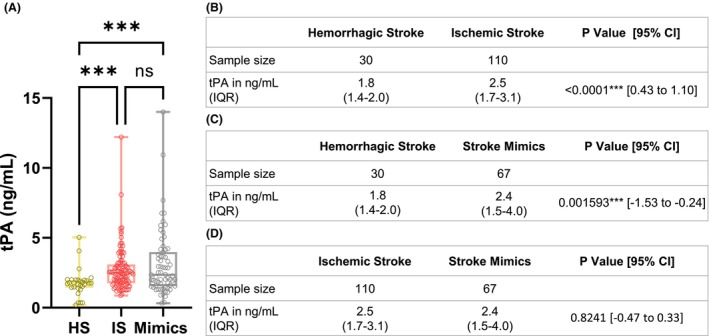
Plasmatic levels of antigenic tPA. Comparison of plasmatic tPA values between hemorrhagic stroke, ischemic stroke and stroke mimics. (A) Box and whisker plots with min., max., 25th, 50th (median) and 75th percentiles. Each circles represent a value for one tPA measurement. Sample size and plasmatic levels of antigenic tPA comparison between hemorrhagic stroke versus ischemic stroke (B); hemorrhagic stroke versus stroke mimics (C); and ischemic stroke versus stroke mimics (D). Data are presented as median (IQR) Mann–Whitney *U* test. CI, confidence interval; IQR, interquartile range; ns, not significant. ***p < 0.001.

The plasmatic levels of total antigenic tPA, assessed within the initial minutes of arrival at the emergency department, revealed noteworthy distinctions exclusively in the context of hemorrhagic strokes, as opposed to both ischemic strokes and stroke mimics (Fig. 2A). Hemorrhagic stroke patients display lower levels of plasmatic tPA compared with both ischemic stroke patients (1.8 ng/mL vs. 2.5 ng/mL, *p* < 0.0001, 95% CI [0.43 to 1.1]; Fig. 2B) and stroke mimics (1.8 ng/mL vs. 2.4 ng/mL, *p* = 0.001593, 95% CI [−1.53 to −0.24]; Fig. 2C). Conversely, no significant differences were observed between ischemic stroke patients and stroke mimics (2.5 ng/mL vs. 2.4 ng/mL, *p* = 0.8241, 95% CI [−0.47 to 0.33]; Fig. 2D).

### Models and logistic regression construction

For our analysis, we designed two models: the “Ambulance model” (referred to as model A) and the “Hospital model” (referred to as model H). The construction of these models is as follows:

- For model A, we consider age, sex, RACE score, and including or not tPA levels in the plasma.
- For model H, we include age, sex, baseline NIHSS score, systolic blood pressure (SBP), and including or not tPA levels in the plasma.

We assessed the efficacy of both models in distinguishing each group (i.e., hemorrhagic strokes, ischemic strokes, and stroke mimics) against the rest (i.e., one‐versus‐rest approach).

### Identification of hemorrhagic strokes within the cohort

**Figure 3:**
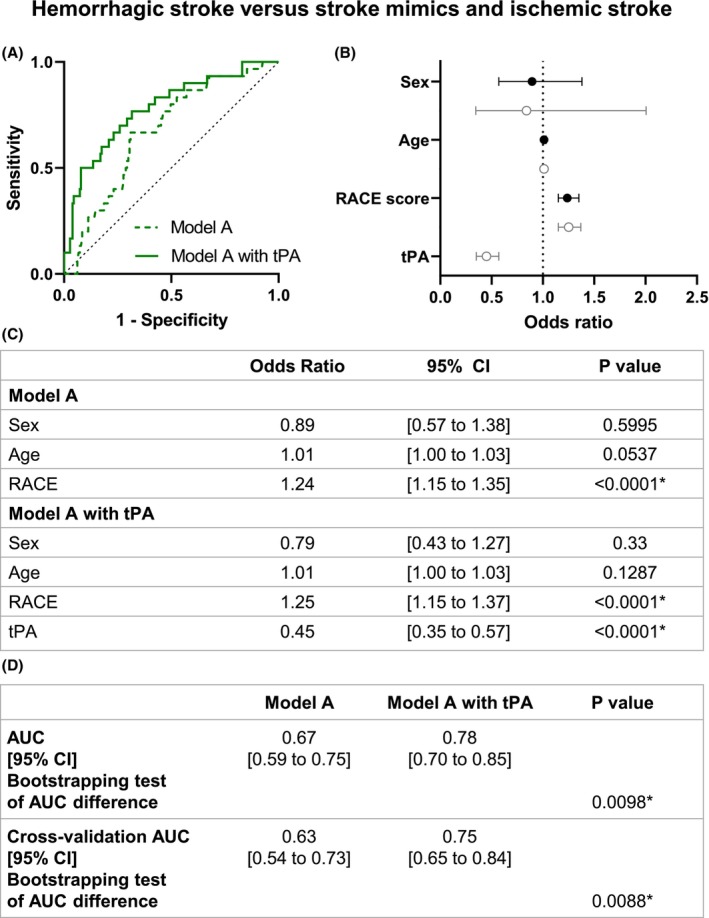
Model A logistic regression of hemorrhagic stroke versus ischemic stroke and stroke mimics patients. (A) ROC curve representation for model A logistic regression with or without tPA levels. The logistic regression models without (AUC = 0.67) and with tPA level (AUC = 0.78) are represented with a continued line and a dotted line, respectively. (B) Logistic regression plot of odds ratios and 95% CI; model A (in black) and model A with tPA (in gray). (C) Models of the logistic regression analyses for comparison between hemorrhagic stroke versus nonhemorrhagic stroke. (D) Statistical comparison of both resulting AUC was performed by bootstrapping model predictions. Model predictions were additionally computed using 10‐fold cross‐validation. AUC, area under curve; CI, confidence interval; RACE, Rapid Arterial oCclusion Evaluation. *p < 0.05.

We first tested if plasmatic tPA levels could increase the accuracy to detect hemorrhagic strokes. The logistic regression model A incorporates sex, age, RACE score, and the presence or not of tPA plasmatic levels (Fig. 3A). In this model, tPA plasmatic levels (OR 0.45, 95% CI [0.35 to 0.57]) and RACE score (OR 1.25, 95% CI [1.15 to 1.37]) were significantly related to clinical status (Fig. 3B,C). The accuracy of the model excluding tPA as a biomarker was 0.67, 95% CI [0.59 to 0.75]. Model accuracy significantly increased to 0.78, 95% CI [0.70 to 0.84] with the inclusion of tPA levels as a predictor (*p* = 0.0098; Fig. 3A,D). Critically, such increase in the model classification accuracy was further confirmed after the internal validation of these results through a 10‐fold cross‐validation: 0.63, 95% CI [0.54 to 0.73] without tPA and 0.75, 95% CI [0.65 to 0.84] with tPA (*p* = 0.0088; Fig. 3D).

**Figure 4:**
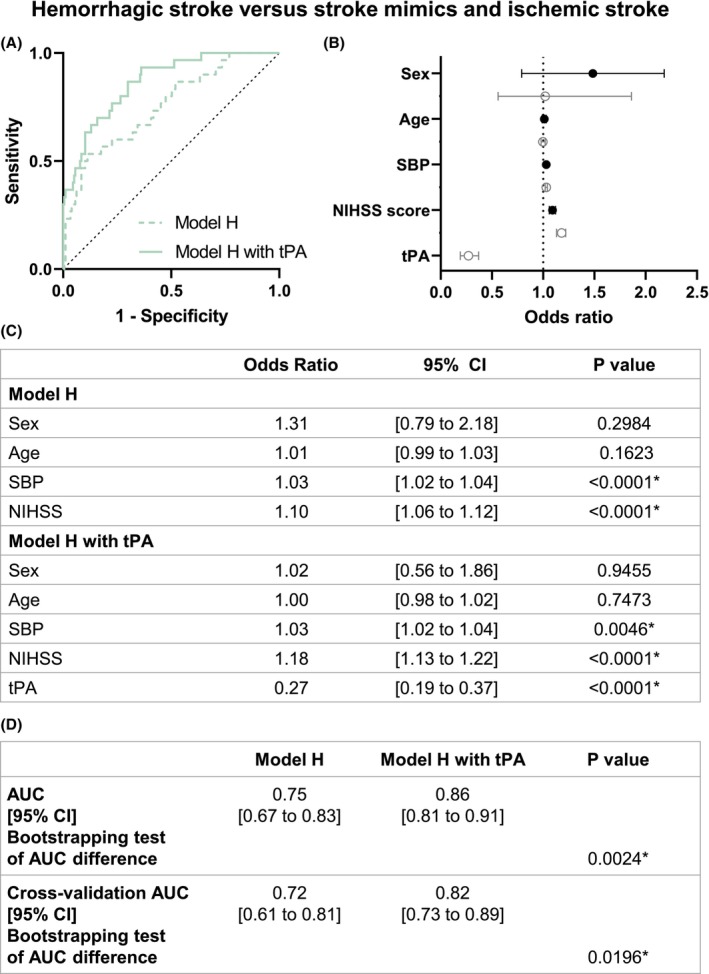
Model H logistic regression of hemorrhagic stroke versus ischemic stroke and stroke mimics patients. (A) ROC curve representation for model H logistic regression with or without tPA levels. The logistic regression models without (AUC = 0.75) and with tPA level (AUC = 0.86) are represented with a continued line and a dotted line, respectively. (B) Logistic regression plot of odds ratios and 95% CI; model H (in black) and model H with tPA (in gray). (C) Models of the logistic regression analyses for comparison between hemorrhagic stroke versus nonhemorrhagic stroke (ischemic stroke and stroke mimics). (D) Statistical comparison of both resulting AUC was performed by bootstrapping model predictions. Model predictions were additionally computed using 10‐fold cross‐validation. AUC, area under curve; CI, confidence interval; NIHSS, National Institutes of Health Stroke Scale; SBP, systolic blood pressure. *p < 0.05.

Model H includes sex, age, SBP, baseline NIHSS, and the presence or not of tPA plasmatic levels (Fig. 4A). In this model, SBP (OR 1.03, 95% CI [1.02 to 1.04]), baseline NIHSS (OR 1.18, 95% CI [1.13 to 1.22]), and tPA levels (OR 0.27, 95% CI [0.19 to 0.37]) exhibited a significant relationship to the clinical status (Fig. 4B,C). Under these conditions, the model's accuracy also improved, increasing from 0.75 (95% CI [0.67 to 0.83]) without tPA to 0.86 (95% CI [0.81 to 0.91]) with tPA (*p* = 0.0024, Fig. 4A,D). Furthermore, the 10‐fold cross‐validation accuracy increased from 0.72 (95% CI [0.61 to 0.81]) without tPA to 0.82 (95% CI [0.73 to 0.89]) with tPA (*p* = 0.0196; Fig. 4D).

### Identification of ischemic strokes within the cohort

**Figure 5:**
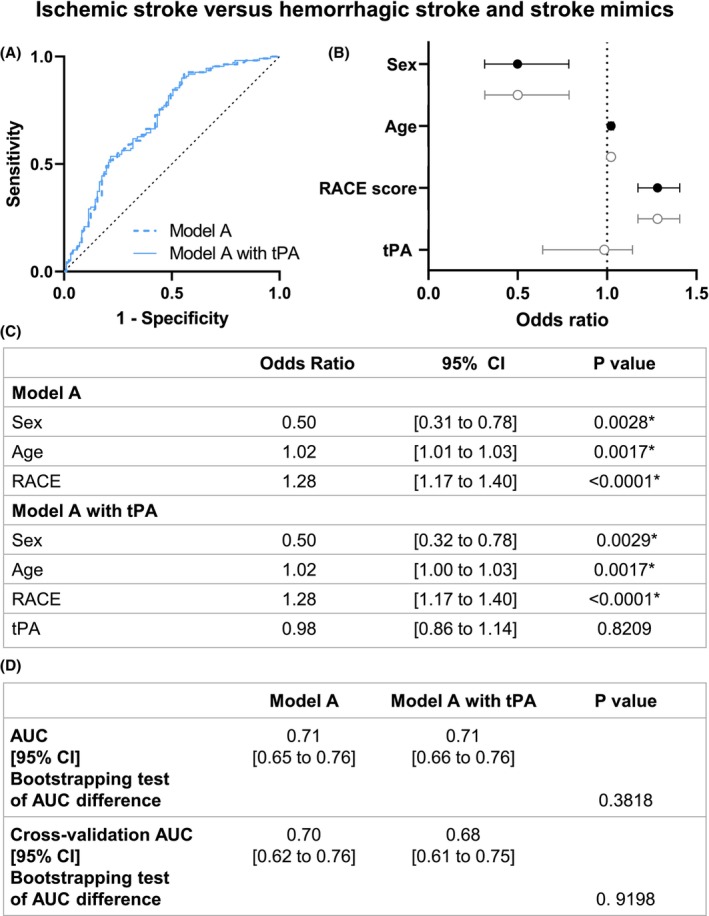
Model A logistic regression of ischemic stroke versus hemorrhagic stroke and stroke mimics patients. (A) ROC curve representation for model A logistic regression with or without tPA levels. The logistic regression models without (AUC = 0.71) and with tPA level (AUC = 0.71) are represented with a continued line and a dotted line, respectively. (B) Logistic regression plot of odds ratios and 95% CI; model A (in black) and model A with tPA (in gray). (C) Models of the logistic regression analyses for comparison between hemorrhagic stroke versus nonhemorrhagic stroke. (D) Statistical comparison of both resulting AUC was performed by bootstrapping model predictions. Model predictions were additionally computed using 10‐fold cross‐validation. AUC, area under curve; CI, confidence interval; RACE, Rapid Arterial oCclusion Evaluation. *p < 0.05.

We then tested if our models were able to discriminate ischemic strokes from hemorrhagic strokes and stroke mimics in model A. Among these included variables, sex (OR 0.5, 95% CI [0.32 to 0.78]), age (OR 1.02, 95% CI [1 to 1.03]), and RACE score (OR 1.28, 95% CI [1.17 to 1.40]) demonstrated a significant association with the diagnosis of ischemic stroke when compared to other types of patients (Fig. 5B,C). The accuracy of the model, excluding tPA as a biomarker, was 0.71, 95% CI [0.65 to 0.76] and was not modified with the inclusion of tPA levels as a parameter (0.71, 95% CI [0.66 to 0.76]; *p* = 0.3818; Fig. 5A,D).

**Figure 6:**
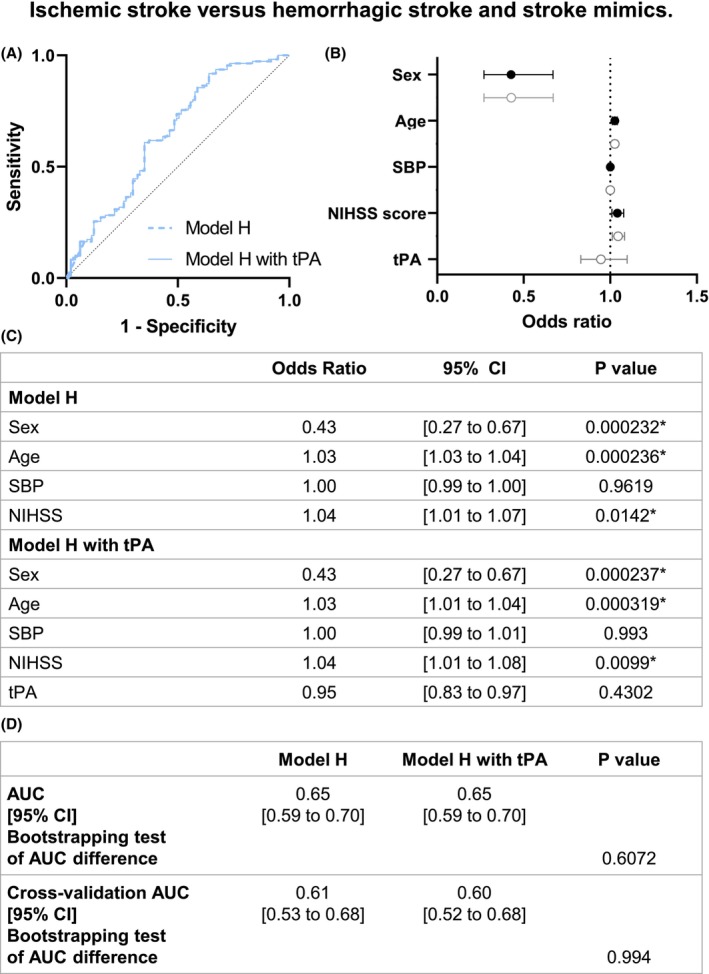
Model H logistic regression of ischemic stroke versus hemorrhagic stroke and stroke mimics patients. (A) ROC curve representation for model H logistic regression with or without tPA levels. The logistic regression models without (AUC = 0.65) and with tPA level (AUC = 0.65) are represented with a continued line and a dotted line, respectively. (B) Logistic regression plot of odds ratios and 95% CI; model H (in black) and model H with tPA (in gray). (C) Models of the logistic regression analyses for comparison between hemorrhagic stroke versus nonhemorrhagic stroke (ischemic stroke and stroke mimics). (D) Statistical comparison of both resulting AUC was performed by bootstrapping model predictions. Model predictions were additionally computed using 10‐fold cross‐validation. AUC, area under curve; CI, confidence interval; NIHSS, National Institutes of Health Stroke Scale; SBP, systolic blood pressure. *p < 0.05.

In model H, sex (OR 0.43, 95% CI [0.27 to 0.67]), age (OR 1.03, 95% CI [1.01 to 1.04]), and baseline NIHSS (OR 1.04, 95% CI [1.01 to 1.08]) are the clinical parameters with significant impact (Fig. 6B,C). As for model A, the discrimination of ischemic stroke compared with other patients was not increased by the inclusion of circulating tPA levels as a biomarker in the model (0.65, 95% CI [0.59 to 0.70] without tPA and 0.65, 95% CI [0.59 to 0.70] with tPA; *p* = 0.6072; Fig. 6A,D).

### Identification of stroke mimics within the cohort

**Figure 7:**
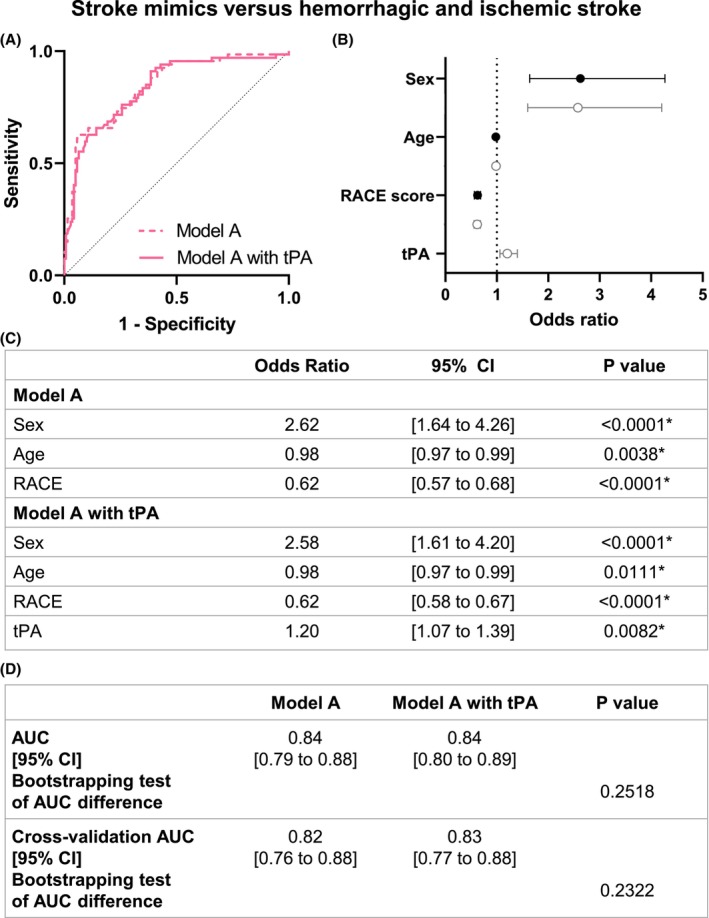
Model A logistic regression of stroke mimics versus hemorrhagic and ischemic stroke patients. (A) ROC curve representation for model A logistic regression with or without tPA levels. The logistic regression models without (AUC = 0.84) and with tPA level (AUC = 0.84) are represented with a continued line and a dotted line, respectively. (B) Logistic regression plot of odds ratios and 95% CI; model A (in black) and model A with tPA (in gray). (C) Models of the logistic regression analyses for comparison between hemorrhagic stroke versus nonhemorrhagic stroke. (D) Statistical comparison of both resulting AUC was performed by bootstrapping model predictions. Model predictions were additionally computed using 10‐fold cross‐validation. AUC, area under curve; CI, confidence interval; RACE, Rapid Arterial oCclusion Evaluation. *p < 0.05.

We finally tested if the inclusion of plasmatic tPA levels in the model increased the discrimination accuracy of stroke mimic against the rest. In model A, sex (OR 2.58, 95% CI [1.61 to 4.2]), age (OR 0.98, 95% CI [0.97 to 0.99]), RACE score (OR 0.62, 95% CI [0.58 to 0.67]), and tPA (OR 1.2, 95% CI [1.07 to 1.39]) were the clinical parameters with significant impact (Fig. 7B,C). The accuracy of the model, excluding tPA as a biomarker, was 0.84, 95% CI [0.79 to 0.88], and was not modified with the inclusion of tPA levels as a parameter (0.84, 95% CI [0.80 to 0.89]; *p* = 0.2518; Fig. 7A,D).

**Figure 8:**
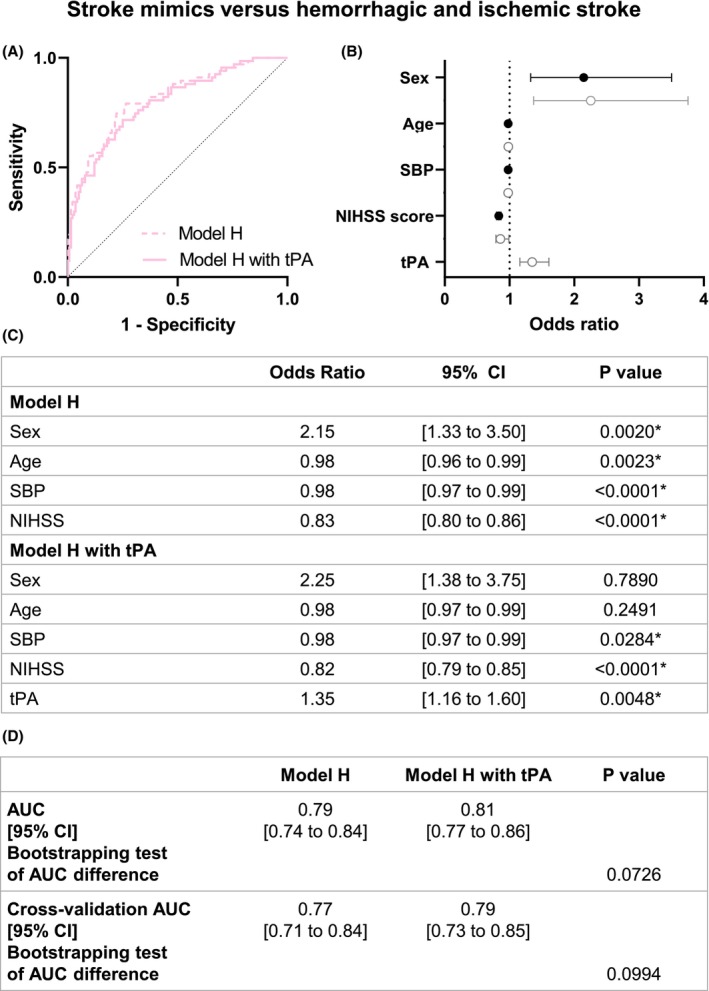
Model H logistic regression of stroke mimics versus hemorrhagic and ischemic stroke patients. (A) ROC curve representation for model H logistic regression with or without tPA levels. The logistic regression models without (AUC = 0.79) and with tPA level (AUC = 0.81) are represented with a continued line and a dotted line, respectively. (B) Logistic regression plot of odds ratios and 95% CI; model H (in black) and model H with tPA (in gray). (C) Models of the logistic regression analyses for comparison between hemorrhagic stroke versus nonhemorrhagic stroke (ischemic stroke and stroke mimics). (D) Statistical comparison of both resulting AUC was performed by bootstrapping model predictions. Model predictions were additionally computed using 10‐fold cross‐validation. AUC: Area under curve. SBP: Systolic blood pressure. NIHSS = National Institutes of Health Stroke Scale. CI: confidence Interval. *p < 0.05.

In model H, SBP (OR 0.98, 95% CI [0.97 to 0.99]), NIHSS (OR 0.82, 95% CI [0.79 to 0.85]), and tPA levels (OR 1.35, 95% CI [1.16 to 1.60]) exhibited a significant relationship with the clinical categories (i.e., mimics or not) (Fig. 8B,C). Yet, the accuracy of Model H was not modified by the addition of tPA (0.79, 95% CI [0.74 to 0.84] vs 0.81, 95% CI [0.77 to 0.86]; *p* = 0.0726; Fig. 8A,D).

## Discussion

We demonstrate that, in contrast to patients with ischemic stroke and stroke mimics, individuals suffering from hemorrhagic stroke exhibit lower levels of tPA. We have developed two models to evaluate the potential of plasmatic tPA as a biomarker for identifying hemorrhagic stroke. The first model, known as “Ambulance” (A), incorporates fundamental parameters that can be assessed by paramedics, including age, gender, and RACE score. The second model, the “Hospital” model (H), employs clinical parameters such as age, gender, SBP (Systolic Blood Pressure), and baseline NIHSS (National Institutes of Health Stroke Scale). In both models, the inclusion of plasmatic tPA levels enhances the accuracy of diagnosing hemorrhagic stroke, thereby establishing it as a valuable biomarker to rapidly detect this condition in a real setting.

In the 90s, circulating tPA has been investigated in coronary pathologies. They discovered a correlation between elevated total tPA levels and an increased risk of cardiovascular pathology.^14^ When it came to ischemic stroke, a retrospective study involving 215 patients revealed that individuals who had recently experienced a stroke exhibited higher circulating levels of both tPA and PAI‐1 (plasminogen activator inhibitor‐1). Importantly, tPA emerged as the primary distinguishing factor in this context.^12^ Interestingly, we could locate just one study that examined tPA levels following a stroke. In this study involving 135 patients and 77 controls, it was found that endogenous tPA was more abundant in ischemic stroke patients. Notably, a logistic regression model demonstrated that tPA antigen levels independently differentiated between ischemic stroke patients and controls.^13^ However, the critical distinction between our study and theirs is the timing of blood collection. While Lindgren and their collaborators obtained blood samples up to 7 days after the stroke (including post‐thrombolysis for some patients), our study collected samples from patients upon their arrival at the hospital, prior to any treatment, all within the initial 24 h following the onset of stroke symptoms.

Nonetheless, our study is subjected to several limitations. Firstly, the Biostroke collection used in this study is retrospective and derived from a single center, which led to some missing data for certain patients. However, the proportion of patients in this bio‐collection reflects the clinical incidence of stroke and stroke suspicions / mimics as seen at the hospital. In order to validate our current findings, it is essential to undertake a prospective observational study involving multiple centers with a larger and more diverse patient sample. Such a study would not only facilitate the collection of additional clinical parameters but also enable the enhancement of the predictive model by incorporating other variables, such as cardiovascular risk factors. This expansion will also allow for a larger cohort of patients to be included. Furthermore, a key bias in this study lies in the utilization of an ELISA kit for biomarker measurement. The total time required for conducting an ELISA experiment is approximately 4.5 h, rendering it unsuitable for an emergency pathology. In order to circumvent this issue, we are actively engaged in the development of a rapid measurement method, capable of quantifying tPA in a mere blood drop within a timeframe of less than 1 hour. Indeed, the quantification method, that is, a laboratory test, needs to be reconsidered for use in clinical/preclinical routines to establish a solid triage method for patients with hemorrhagic stroke. The approach varies depending on the country, as legislation and medical recommendations for stroke suspicions differ. While some countries send a medical doctor to the patient, others only send paramedics. Additionally, in some countries, paramedics are not authorized to perform biological tests or draw blood. Our ultimate objective is to standardize the process from a single blood drop, similar to a glycemic test, to conduct a European multicentric study.

We are aware that our test will not replace a CT scan or MRI; however, it could save time for patients in many situations. While drug‐mediated thrombolysis for ischemic stroke can be performed in most hospitals in industrialized countries, hemorrhagic stroke requires neurosurgery and intensive care units that are typically available only in larger hospitals. A test that could identify hemorrhagic stroke patients in distant or rural areas could prevent the need for secondary transport by directing them to the appropriate hospital initially. This test could also be beneficial in countries with limited healthcare systems, where CT scans are not readily available or are too expensive.

Nevertheless, we clearly have a proof of concept that using total antigenic tPA as a biomarker within the ambulance, associated to simple parameters such as sex, age, and RACE score, would facilitate the identification, and thus the orientation, of hemorrhagic stroke patients. This became crucial following the findings of the randomized controlled trial, INTERACT3, which involved 7036 patients. The trial demonstrated that the adoption of a care bundle protocol aimed at reducing blood pressure during ambulance transport led to improved outcomes for patients experiencing acute intracerebral hemorrhage.^15^

In conclusion, the use of tPA as a biomarker holds promise as a valuable tool for identifying hemorrhagic strokes in case of stroke suspicion (including ischemic and mimics), both in pre‐hospital settings for patient triage and within hospital management. This can open a new strategy for addressing patients from rural areas to the dedicated hospital, or for patients from countries where CT scan or MRI is not readily available or is prohibitively expensive. However, as outlined earlier, the development of a rapid and “easy to use” test is of utmost importance to validate our findings through a multicenter prospective observational study.

## Funding Information

The study was funded by the INSERM, Normandie University, and the Normandie Region.

## Authors Contribution

MJ, EP, AMT, DV, EL, RM, and BDR contributed to the conception and design of the study. MJ, PG, EL, AR, HL, BL, MB, VR, EL, RM, and BDR contributed to acquisition and analysis of data. MJ, PG, JM, BC, DV, EL, RM, and BDR contributed to drafting the text and/or preparing the figures.

## Conflict of Interest

The authors declare no conflicts of interests.

## Data Availability

The data that support the findings of this study are available on request from the corresponding author. The data are not publicly available due to privacy or ethical restrictions.
